# Human herpesvirus multiplex ddPCR detection in brain tissue from low- and high-grade astrocytoma cases and controls

**DOI:** 10.1186/s13027-016-0081-x

**Published:** 2016-07-26

**Authors:** Cheng-Te Major Lin, Emily C. Leibovitch, M. Isabel Almira-Suarez, Steven Jacobson

**Affiliations:** 1 The National Institute of Neurological Disorders and Stroke, National Institutes of Health, BG 10 RM 5C103 10 Center Dr., Bethesda, MD 20892 USA; 2 School of Medicine and Health Sciences, The George Washington University, Ross Hall 2300 Eye Street, NW, Washington, DC 20037 USA

**Keywords:** CMV, HHV-6A, HHV-6B, EBV, Herpesvirus, Astrocytoma, Glioblastoma, ddPCR

## Abstract

**Background:**

Glioblastoma (GBM) is a fatal CNS malignancy, representing 50 % of all gliomas with approximately 12–18 months survival time after initial diagnosis. Recently, the human herpesvirus cytomegalovirus (CMV) has been suggested to have an oncogenic role, yet this association remains controversial. In addition, human herpesvirus 6 (HHV-6) and Epstein-Barr virus (EBV) have also been associated with low-grade gliomas, but few studies have examined HHV-6 and EBV in glioblastomas. Droplet digital PCR (ddPCR) is a highly precise diagnostic tool that enables the absolute quantification of target DNA. This study examines the association between multiple human herpesviruses and astrocytomas.

**Methods:**

This study analyzed 112 brain tissue specimens, including 45 glioblastoma, 12 astrocytoma grade III, 2 astrocytoma grade II, 4 astrocytoma grade I, and 49 controls. All brain tissue samples were de-identified and pathologically confirmed. Each tissue block was sectioned for DNA extraction and CMV, EBV, HHV-6A and HHV-6B, and a cellular housekeeping gene were amplified by ddPCR.

**Results:**

Neither CMV nor HHV-6A were detected in any of the astrocytoma samples. However, HHV-6B (*p* = 0.147) and EBV (*p* = 0.049) had a higher positivity frequency in the GBM compared to the controls.

**Conclusion:**

The undetectable CMV DNA in the astrocytoma cohort does not support the observation of an increased prevalence of CMV DNA in GBM, as reported in other studies. EBV has a significantly higher positivity in the GBM cohort compared to the controls, while HHV-6B has a higher but not statistically significant positivity in the case cohort. Whether these viruses play an oncogenic role in GBM remains to be further investigated.

## Background

Gliomas are brain tumors that develop from glial cells. Approximately a quarter of diagnosed gliomas are astrocytomas, which are scaled from grade I to grade IV based on histological features [1]. According to the World Health Organization (WHO) classification for CNS tumors: astrocytoma grade I and astrocytoma grade II are classified as low-grade astrocytomas, while astrocytoma grade III and astrocytoma grade IV are classified as high-grade astrocytomas. Furthermore, astrocytoma grade IV is also referred to as glioblastoma (GBM). Patients with GBM have a median survival of 12 months and an average 5-years survival rate less than 5 % [2, 3]. Although GBM has been extensively studied in clinical and laboratory settings, there are limited effective treatments for this malignant tumor [4].

The herpersviruses are large, enveloped, double-stranded DNA viruses. Currently, there are more than 100 known herpesviruses, and nine human herpesviruses (HHVs) that can cause a wide range of diseases. The β-herpesvirus subfamily, one of three herpesvirus subfamilies, has been suggested to be associated with central nervous system (CNS) impairments and diseases, such as hearing loss, CNS inflammation, and primary brain tumors. Recently, accumulating evidence indicates that cytomegalovirus (CMV), a β-herpesvirus, is associated with GBM, but these reports remain controversial [5, 6]. For example, some studies have demonstrated CMV viral genome expression in glioblastoma samples compared to other types of non-neurological disease patients, whereas other have found no trace of CMV DNA in glioblastoma. The discrepancy between these findings may be contributed by differences in the experimental methodology, brain tissue specimen preservation, and DNA detection methods [7]. The vast majority of studies were done by PCR or immunohistochemistry, which focused on the molecular identification of viral DNA or viral proteins, respectively. Such discrepancy encourages a more in-depth investigation of the role of CMV and its association with glioblastoma.

Another human β-herpesvirus, human herpesvirus 6 (HHV-6) has also been associated with low-grade gliomas [8–10], but few studies have examined HHV-6 in glioblastomas. There are two species of HHV-6, HHV-6A and HHV-6B. Both viruses differ in their epidemiological, biological, and molecular properties. Less is known about the specificity of HHV-6A-associated diseases compared with HHV-6B, which is the etiologic agent of roseola, and associated with CNS diseases such as status epilepticus, mesial temporal sclerosis, and multiple sclerosis [11–13]. Another subfamily of human herpesviruses, the γ-herpesviruses, establishes latent infection in B-lymphocytes and may cause these cells to proliferate abnormally, leading to Kaposi’s sarcoma and CNS lymphoma [14]. Epstein-Barr virus (EBV) is thought to be associated with specific oncogenic conditions, such as Hodgkin’s lymphoma, Burkitt’s lymphoma, nasopharyngeal carcinoma and a subset of gastric carcinomas [15]. EBV genomic DNA has also been detected in high-grade glioma samples by unmapped next-generation sequencing, but mechanisms of pathogenesis are still unclear [16]. Overall, the extent to which human herpesviruses are associated with GBM is currently unknown.

More recently, a randomized and blinded clinical trial examined the effectiveness of a CMV vaccine (CMV pp65 RNA pulsed DCs) with different pre-conditioning regimens for treating GBM. Aside from identifying an immunogenic preconditioning regimen, the study demonstrated that an increase in CMV pp65-specific immune responses correlated with overall GBM survival [17]. Although these clinical trials have shown promising results regarding positive clinical outcomes for GBM patients, the significance of CMV in GBM remains controversial [18]. However, this study provides clinical evidence that anti-viral therapies can improve patient outcomes, which suggests that viruses, specifically herpesviruses, might play a role in these malignant brain tumors.

To investigate the presence of herpesvirus genome sequences in different grades of astrocytomas and controls, we utilized a novel methodology to detect and quantify multiple human herpesviruses. Droplet digital PCR (ddPCR) is a newly developed technology that divides PCR reactions into tens of thousands droplets and detects the amplification in each droplet, which enables us to detect and quantify target DNA directly [19, 20]. Other studies have used this highly precise technology to detect bacterial or viral genomic DNA sequences in various types of samples [21–23]. To quantify multiple human herpesviruses in astrocytoma brain tissue specimens and non-neurological disease control specimens, we developed a multiplexing method, which enables the simultaneous screening of multiple target sequences in brain tissue samples.

This study quantitated CMV, HHV-6A, HHV-6B and EBV viral DNA in astrocytoma (WHO grade I-IV) and non-neurological disease control tissues with a newly developed ddPCR multiplexing method.

## Methods

### Study design

This was a retrospective and health center-based study. The study screened for multiple human herpesviruses (CMV, HHV-6A, HHV-6B, and EBV) in different grades of astrocytomas and non-neurological disease controls, which excluded other brain cancers and other CNS diseases.

Samples were obtained from the George Washington University Hospital and the National Institutes of Health, and the protocol was approved by both institutional review boards. The study collected specimens preserved by three different methods: formalin-fixed paraffin embedded (FFPE), optimal-cutting temperature (OCT), and fresh frozen. Study brain tissue specimens were selected based on medical records from the institutions. A total of 112 unique adult brain tissue samples were included in the study. The study collected astrocytomas (WHO grades I-IV) and non-neurological disease controls. The astrocytoma FFPE cases were comprised of four grade I, one grade II, two grade III, and 19 grade IV (GBM). Also, there were ten grade III astrocytomas and 20 grade IV (GBM) collected as the OCT biopreserved tissue. Lastly, there was a total of six fresh frozen astrocytomas grade IV (GBM) and one fresh frozen astrocytoma grade II. Non-neurological disease tissue specimens were selected based on medical records from both institutes. These controls were obtained from autopsy tissue specimens, which were not associated with neurological disease as cause of death. There were 49 non-neurological disease brain specimens, comprised of 32 fresh frozen samples and 17 FFPE-preserved samples (Table 1). The fresh frozen control samples were from the cerebellum and parietal regions of the brain, whereas the FFPE-preserved control samples were from the left fronto-parietal cortex. The adult control cohort had an average age of 51.7 years old with 20 females and 29 males. The astrocytoma (WHO grades I–IV) tissue specimens had an average age of 35 years old. After the genomic DNA extraction, the samples were screened by ddPCR for multiple human herpesviruses based on highly conserved genomic sequences. Cases classified by different astrocytoma WHO grades were compared with the non-neurological disease controls with regard to human herpesviruses positivity frequency and viral loads.

**Table 1:** Age, sex, cause of death for non-neurological disease controls (*n* = 49)

	Age (years)	Sex	Cause of death
Fresh Frozen Tissue			
	33	M	Coronary Artery Thrombosis
	34	F	Asthma
	64	M	ASCVD^a^
	50	M	ASCVD
	34	M	Abdominal Injuries
	48	M	HASCVD^b^
	64	F	ASCVD
	43	F	HASCVD
	30	M	Multiple Injuries
	34	M	HASCVD
	40	F	Multiple Injuries
	43	F	HASCVD
	52	M	ASCVD
	49	M	Pulmonary Thromboembolism
	59	M	Bronchopneumonia
	40	F	Pulmonary Embolism
	28	M	ARVC^c^
	53	M	HASCVD
	44	M	HASCVD
	50	F	HASCVD
	35	F	Streptococcal Sepsis
	48	F	HASCVD
	37	M	ASCVD
	63	F	Acute Myocardial Infarction
	23	M	Hypertrophic Cardiomyopathy
	56	F	Asthma
	27	M	ASCVD
	57	M	ASCVD
	54	F	ASCVD
	63	M	HASCVD
	36	M	ASCVD
	60	F	HASCVD
FFPE-Preserved Tissue			
	74	F	Breast Cancer
	64	M	Lipoid Pneumonia
	56	M	Ascending Aortic Dissection
	43	M	Acute Pneumonia
	64	F	Acute Ischemic Bowel Disease
	63	F	Congestive Heart Failure
	65	M	Laryngeal Squamous Cell Carcinoma
	86	M	Acute Abdominal Compartment Syndrome
	41	M	*P. aeruginosa* Sepsis
	68	F	Acute Fibrinous and Organizing Pneumonia
	67	M	Acute Myocardial Infarction
	55	M	Subacute Bacterial Endocarditis
	66	M	Mallory Weiss Tear
	84	F	Sudden Cardiac Death
	49	F	Sickle Cell Disease
	65	F	Acute Myocardial Infarction
	70	M	Congestive Heart Failure

^a^ Atherosclerotic Cardiovascular Disease

^b^ Hypertensive Arteriosclerotic Cardiovascular Disease

^c^ Arrhythmogenic right ventricular cardiomyopathy

### Brain tissue DNA extraction

#### Formalin-Fixed Paraffin Embedded (FFPE)

FFPE brain tissue genomic DNA was extracted using the QIAamp DNA FFPE Tissue kit (Qiagen, Valencia, CA). A total of 500 μm (ten sections 50 μm thick) of FFPE tissue was used in the genomic DNA extraction, which was performed according to the manufacturer’s specifications. Samples were eluted with 100 μl of buffer AE.

#### Optimal-Cutting Temperature (OCT)

OCT brain tissue genomic DNA was extracted using the QIAamp DNA Mini kit (Qiagen, Valencia, CA). A total of 500 μm (ten sections 50 μm thick) of OCT tissue was used in the genomic DNA extraction. The OCT compound was dissolved in 1 ml of PBS (Ca-Mg free) and spun at 8000 rpm for 5 min. PBS washes were repeated if the samples had significant remaining amounts of OCT. Samples were eluted with 100 μl of buffer AE.

#### Fresh frozen

Fresh frozen brain tissue genomic DNA was extracted by the DNeasy Blood and Tissue kit (Qiagen, Valencia, CA). A total of 25 mg of fresh frozen brain tissue was used in the genomic DNA extraction, performed according to the manufacturer’s instructions. Samples were eluted with 100 μl of buffer AE.

### Multiplex digital droplet PCR assay

The CMV, EBV, and HHV-6 primers were selected from highly conserved regions, *ul55*, *lmp1*, and *u57*, respectively (Table 2) [24–27]. Different probes were used to distinguish between HHV-6A (FAM) and HHV-6B (VIC), as described by Leibovitch et al. in 2014 [25]. The 20X ddPCR primer/probe solution was composed of 18 μl of 100 μM forward primer, 18 μl of 100 μM reverse primer, 5 μl of 100 μM probe (FAM or VIC), and 79.5 μl PCR quality distilled water. With the multiplexing method, the 10X ddPCR primer/probe solution was composed of 9 μl of 100 μM forward primer, 9 μl of 100 μM reverse primer, 2.5 μl of 100 μM probe (FAM or VIC), and 59 μl PCR quality distilled water. The ddPCR primer/probe concentration difference generated distinct populations on the Y-axis. HHV-6A, HHV-6B, and *rpp30* were detected with the 20X ddPCR mastermix solution; meanwhile, CMV and EBV were detected with the 10X ddPCR mastermix solution. Each plot displayed the viruses or the housekeeping gene (*rpp30*) populations in a brain tissue specimen. *rpp30* (NCBI gene 10556) is an housekeeping gene, which was used as a human genome reference loci, and served as an indicator of brain tissue DNA quality and cellular quantity. A specimen was considered positive when the 2-D fluorescence plots showed at least two dots at the expected amplitude. Each multiplex positive sample was further confirmed by a  singleplex ddPCR assay, which targeted only one genomic sequence per axis. Samples were considered positive only if both multiplex and singleplex assays showed equal or more than two droplets at the expected amplitude. More detailed multiplex ddPCR method is provided by the Bio-Rad ddPCR guidelines [20]. The viral load calculation and statistical analysis were based on the multiplex ddPCR assay.

**Table 2:** ddPCR assay primers and probes

	Forward primer (5′-3′)	Reverse primer (5′-3′)	Probe
HHV-6A *u57*	CCGTGGGATCGTCTAAAATTATAGATGT	CCACACTAGTCCGGACGGATAA	FAM – CTGGAACTGTATAATAGG – MGBNFQ
HHV-6B *u57*	CCGTGGGATCGTCTAAAATTATAGATGT	CCACACTAGTCCGGACGGATAA	VIC – CTGGAGCTGTACAACAG – MGBNFQ
CMV *ul55*	TGGGCGAGGACAACGAA	TGAGGCTGGGAAGCTGACAT	FAM – TGGGCAACCACCGCACTGAGG – 3BHQ
EBV *lmp1*	AAGGTCAAAGAACAAGGCCAAG	GCATCGGAGTCGGTGG	FAM – AGCGTGTCCCCGTGGAGG – MGBNFQ
*RPP30* ^*a*^	GATTTGGACCTGCGAGCG	GCGGCTGTCTCCACAAGT	VIC – CTGACCTGAAGGCTCT – MGBNFQ

^a^ Ribonuclease P protein subunit p30

### Statistical analysis

With the retrospective study design, we computed the positivity frequency proportions in cases and controls to examine the proportional differences between each human herpesvirus and different grades of astrocytoma or controls. *p*-value at 95 % confidence level is presented in each group. Based on the Fisher’s Exact test, we included the *p*-value to determine statistically significant proportions between cases and controls. We used RStudio software 0.99.486 version developed by RStudio, Inc. and Prism 6 developed by GraphPad software, Inc to analyze the data.

## Results

### ddPCR multiplex assay characterization

**Fig. 1:**
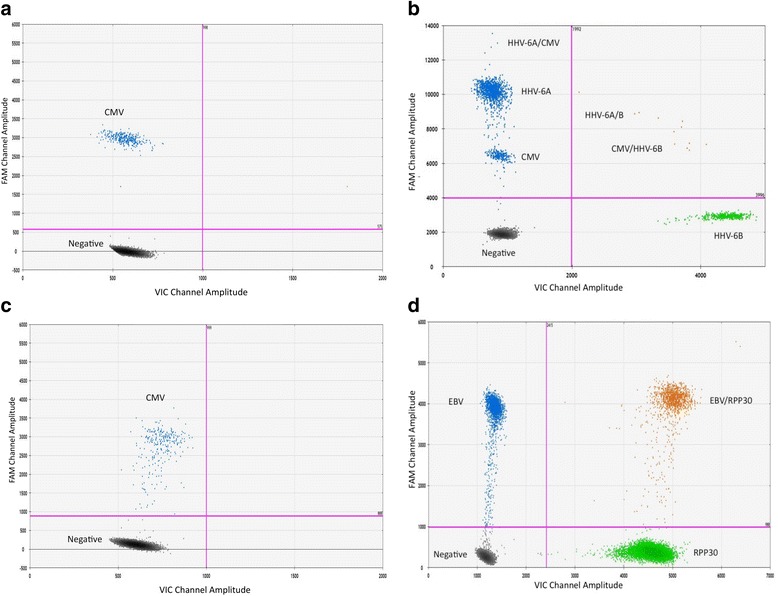
Characterization of the HHV-6A, HHV-6B, CMV, EBV and RPP30 multiplex ddPCR assays. A CMV plasmid was used as a positive control to evaluate each CMV primer/probe set, with results shown in (**a** and **b**). **a** The ddPCR-adapted CMV (20X) primer/probe set used in this study [24] is shown on the FAM channel; **c** A second ddPCR-adapted CMV (20X) primer/probe set [28] that was evaluated but not used in this study is shown on the FAM channel; **b** HHV-6A and HHV-6B infected cells were used as positive controls in this multiplex characterization. HHV-6A (20X) and CMV (10X) are on the FAM channel and HHV-6B (20X) is on the VIC channel. Droplets containing multiple DNA targets are double positive for FAM and VIC (shown in the upper right quadrant); **d** An EBV transformed lymphoblastoid cell line was used as a positive control in this multiplex characterization. EBV is shown on the FAM channel and the housekeeping gene RPP30 is shown on the VIC channel

The present study optimized the CMV ddPCR assay by comparing three published CMV primers and probe sets [24, 28, 29]. After adjusting the published sequences to fit ddPCR parameters, one primer/probe set had a high GC content that impeded efficient amplification of target sequences [29]. Therefore, two CMV primer/probe sets were compared for sensitivity and performance in the ddPCR assay (Fig. 1a and c). Though both detected CMV positivity in the positive control, one set generated a tighter, higher amplitude positive population (Fig. 1a), suggesting greater target DNA affinity compared to the other CMV primer/probe set, which generated a more scattered positive population (Fig. 1c). Therefore, the ddPCR-adapted CMV primer/probe set shown in Fig. 1a was used for this study and multiplexed with HHV6A, HHV6B, and EBV. The HHV-6A and HHV-6B primer/probe sets were described in a recent study of coinfection [25]. The EBV primer/probe set was adapted from published sequences [27] and optimized to fit ddPCR-specific parameters (unpublished data). The results in Fig. 1c and d are representative of these combinations. Clear separations can be seen with HHV-6A and CMV on the FAM channel (Y-axis) and HHV-6B on the VIC channel (X-axis) (Fig. 1b). Likewise, distinct populations are seen with EBV on the FAM channel (Y-axis) and RPP30 on the VIC channel (X-axis) (Fig. 1d). These data show that successful herpesvirus multiplex ddPCR can be achieved, with detection sensitivities that are comparable to singleplex ddPCR (data not shown) [24, 25].

### Undetectable CMV viral genome sequences in GBM

**Fig. 2:**
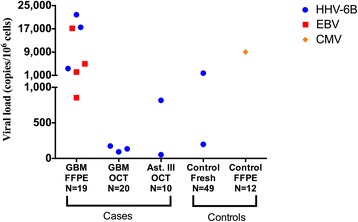
Viral loads of HHV-6B, EBV and CMV in PCR positive high-grade astrocytoma cases and controls. Viral loads are expressed as viral copies per million cells, and stratified by tissue type (cases or controls) and preservation method. Only positive results are shown. *Blue circles* correspond to HHV-6B positive samples, *red squares* correspond to EBV positive samples, and *orange diamonds* correspond to CMV positive samples. FFPE – formalin fixed paraffin embedded; OCT – optimal cutting temperature; Ast. III – Astrocytoma Grade III; GBM – Glioblastoma

Each astrocytoma case was screened for CMV by both the multiplex and singleplex ddPCR assays. All clinical tissue specimens were collected either as FFPE, OCT, or fresh frozen biopreservation methods. Within each astrocytoma case cohort and non-neurological disease control cohort, there was no frequency difference in CMV detection by ddPCR assay between these preservations methods. Furthermore, no CMV reactivity was observed in any tissue specimens when assayed by either a singleplex or multiplex ddPCR. As shown in Table 3, the present study did not detect CMV genomic DNA in any astrocytoma case (*n* = 63) with either ddPCR assay, although one FFPE non-neurological disease tissue specimen showed CMV positivity with a viral load of 9023 copies/10^6^ cells (Fig. 2). Collectively, the absence of CMV DNA in our GBM cohort does not support an association of CMV and glioblastoma.

**Table 3:** Positivity frequency of four human herpesviruses across all brain specimens

		CMV	HHV-6A	HHV-6B	EBV
FFPE-Preserved Tissue					
	Astrocytoma Grade IV (*n* = 19)	0/19	0/19	3/19 (15.8 %)	4/19 (21.1 %)
	Astrocytoma Grade III (*n* = 2)	0/2	0/2	0/2	0/2
	Astrocytoma Grade II (*n* = 1)	0/1	0/1	0/1	0/1
	Astrocytoma Grade I (*n* = 4)	0/4	0/4	0/4	0/4
	Control (*n* = 17)	1/17 (5.9 %)	0/17	0/17	0/17
OCT-Preserved Tissue					
	Astrocytoma Grade IV (*n* = 20)	0/20	0/20	3/20 (15 %)	0/20
	Astrocytoma Grade III (*n* = 10)	0/10	0/10	2/10 (20 %)	0/10
Fresh Frozen Tissue					
	Astrocytoma Grade IV (*n* = 6)	0/6	0/6	0/6	0/6
	Astrocytoma Grade II (*n* = 1)	0/1	0/1	0/1	0/1
	Control (*n* = 32)	0/32	0/32	2/32 (6.2 %)	0/32

### High HHV-6B positivity frequency in astrocytomas

**Fig. 3:**
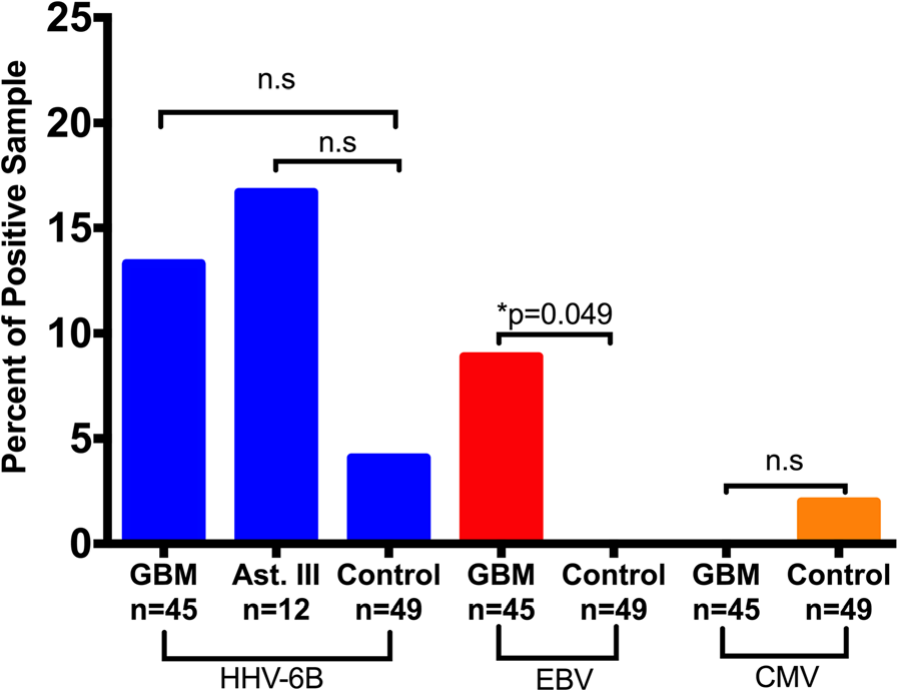
Positivity frequency of HHV-6B, EBV and CMV across high-grade astrocytoma cases and controls. Percent frequency of detected HHV-6B, EBV, and CMV across all biopreservation methods, stratified by tumor grade (GBM and astrocytoma grade III) compared to non-neurological disease controls. Statistical significance is defined as *p* < 0.05 C.I 95 % (n.s – not significant). FFPE – formalin fixed paraffin embedded; OCT – optimal cutting temperature; Ast. III – Astrocytoma Grade III; GBM – Glioblastoma

By contrast with CMV, HHV-6B was detected at a relatively high frequency in the brain tissue specimens compared to the other human herpesviruses investigated in this study (HHV-6A, CMV, and EBV). Figure 2 shows HHV-6B, EBV and CMV viral loads in each brain tissue specimen. HHV-6B was the most frequently detected virus among the brain specimens, with viral loads ranging from less than 100 to greater than 20,000 copies per million cells. HHV-6B was detected in 3/19 (15.8 %) FFPE GBM samples, 3/20 (15 %) OCT GBM samples, 2/10 (20 %) OCT astrocytoma grade III, and 2/32 (6.2 %) fresh frozen control samples (Table 3). However, HHV-6B was also detected in a low number of brain tissues from non-neurological disease controls (2/49, 4.1 %), and was therefore not statistically significant in the GBM cohort (*p* = 0.147). HHV-6B was also detected in 2/10 (20 %) astrocytoma grade III samples, but again the proportion compared to controls was not statistically significant (*p* = 0.170) (Fig. 3). HHV-6A was not detected in any of the astrocytoma or non-neurological disease tissue specimens. HHV-6 is a ubiquitous human herpesvirus in the adult population and the CNS has been suggested as a site of viral latency and reactivation, which is consistent with the HHV-6B positivity observed in our non-neurological disease control cohort [30].

### Significantly higher proportion of EBV in GBM

While high levels of HHV6B could be demonstrated in high-grade astrocytoma cases (grade III and IV), EBV was only detected in the FFPE GBM samples, 4/19 (21.1 %) and not in any controls (Fig. 3 and Table 3) (*p* = 0.049). These samples had EBV viral loads ranging from less than 1000 to greater than 17,000 copies per million cells (Fig. 2). Most cases did not show co-infection (two or more viruses) by the multiplex ddPCR assay, however two FFPE GBM tumor specimens were positive for both HHV-6B and EBV. Both cases were verified by both multiplex and singleplex assays. Collectively, the results in this study demonstrated that HHV-6B and EBV, but not CMV,  could be amplified from high-grade astrocytomas using a human herpesvirus multiplex ddPCR.

## Discussion

In recent years, many studies of herpesviruses and brain tumors have examined the association between CMV and high-grade astrocytomas, but the relationship is still controversial [31]. Also, no previous study has systematically examined CMV, HHV-6A, HHV-6B, and EBV viral DNA in low- and high-grade astrocytomas. The human herpesviruses have the ability to become latent in specific host cell types, primarily in the CNS and lymphoid tissue [32]. A review of recent studies that used immunohistochemistry (IHC), in situ hybridization (ISH), and PCR to detect CMV in GBM tumor specimens showed that results vary from undetected to 100 % positivity [18]. IHC and PCR each focus on different molecular targets, so detection selectivity discrepancies might be contributing to discordant results. Both techniques have their respective advantages and disadvantages. For instance, the interpretation of IHC is complicated due to background pigments, such as hematoidin, hemosiderin, formalin precipitates, and unspecific staining [5]. While PCR only focuses on a selected genomic sequence, off target amplification may result in false positives, and negative PCR results, i.e. the non-amplification of a specific amplicon, cannot be interpreted as the absence of the viral genome.

The absence of CMV in the GBM samples in this present study does not support a CMV genomic DNA and glioblastoma association, in contrast to other studies that have reported a high prevalence of CMV genomic DNA in glioblastoma by PCR [33–35]. Most studies have  utilized either a protein or a genomic sequence targeting technique, but not both, to detect CMV. A few studies have proposed that certain onco-viruses might trigger viral DNA recombinogenic activities, which could promote oncogenesis with the secondary loss of the viral genome in tumor cells [36, 37]. Viruses that initiate such “hit and run oncogenesis” in the host may be absent at the time of screening.

Other than screening for known viral sequences or using viral-specific IHC assays, a less biased approach would be to sequence brain tumors and search for viral or non-human nucleic acid sequences, for example using a next-generation sequencing platform [38]. With this approach, a report in 2014 demonstrated EBV and HHV-6 viral sequences in 5/21 (24 %) and 1/21 (5 %) of GBM cases, respectively [16]. These viral positivity levels are consistent with the results of this present study, which detected EBV and HHV-6B in 4/45 (8.9 %) and 6/45 (13.3 %) of GBM cases, respectively. However, since the 2014 study did not investigate a control cohort, this limited the interpretation of the roles of EBV and HHV-6 in their GBM cohort.

Although to our knowledge no studies have specifically investigated HHV-6B in glioblastoma, some studies have suggested the involvement of HHV-6 in patients with gliomas. A 2012 study reported 17 of 40 (42.5 %) glioma samples with detectable HHV-6, compared to one of 13 (7.7 %) controls, using nested-PCR [8]. Furthermore, among the glioma cohort, 7/14 GBM (50 %) were HHV-6 positive. In a separate 2009 study, Crawford and colleagues reported a three-fold increase in HHV-6 positivity by IHC in glial tumors compared to non-glial tumors in an adult cohort, and 14/30 (46.7 %) of CNS tumors were HHV-6 positive by nested-PCR compared to 0/25 control brains [39]. While these data suggest the involvement of HHV-6 in at least a subset of brain tumors, the specific potential oncogenic mechanisms of HHV-6 are not fully understood.

We found EBV to be present at a significantly higher frequency in GBM compared to controls. EBV is an oncovirus that is responsible for the development of Hodgkin’s lymphoma, nasopharyngeal carcinoma and Burkitt’s lymphoma. However, few studies have examined the relationship between EBV and astrocytomas, specifically. In a study by Neves et al. in 2012, EBV was the most frequently detected human herpesviruses in pilocytic astrocytoma (WHO grade I) by PCR, but at fairly low levels (<100/100 ng cellular DNA) [40]. Neves and colleagues detected only one sample with CMV, but did not detect any HHV-6. Further epidemiological and molecular studies are needed to investigate the role of EBV in astrocytomas.

Among the glioblastoma cohort in this present study, there were two cases with co-infection of EBV and HHV-6B. Currently, no study has reported EBV and HHV-6 co-infection in glioblastoma, though this has been observed in suspected viral CNS disease samples [41, 42]. Several in-vitro studies indicate that HHV-6 may activate EBV expression, which might promote EBV-associated diseases later in life [43, 44]. A 2015 study reported two encephalitis cases with HHV-6 and EBV co-infection detected by PCR [45]. However, the coinfection of these two herpesviruses in our case cohort cannot be seen as causal agents of the astrocytoma. The detection of HHV-6B and EBV could be a mere consequence of molecular events within the tumor that may be associated with reactivation of latent virus [46].

In our cohort, the sensitivity of multiple human herpesvirus detection did not vary with the biopreservation method. All preservation methods were comparable in terms of virus detection, though fresh-frozen remains the most ideal for viral detection. Some studies have raised concerns about the possibility of DNA degradation upon long-term storage in FFPE. The vast majority of studies used FFPE-preserved tissues for the detection of viral protein or DNA. However, in a study that examined the quality of FFPE extracted DNA, no significant differences were found between DNA extracted from FFPE tissue blocks that had been stored for less than one year and FFPE tissue blocks that had been stored for 1–11 years [47].

Lastly, the ddPCR multiplex assay is a highly precise viral diagnostic tool that enables the absolute quantification of multiple viral target regions [48, 49]. Although multiplex qPCR has a shorter analysis time, high sensitivity, and a broad dynamic range, the multiplex ddPCR assay has lower variability, outstanding accuracy, and provides better quality of quantification in the multiplex assay [49]. A study reported by Hayden in 2013 compared quantitative real-time PCR (qRT-PCR) and ddPCR for the detection of CMV in clinical samples, and reported that ddPCR showed less variability when detecting samples at higher concentrations [50]. Overall, the study indicated that ddPCR is an accurate assay for detecting CMV viral loads in clinical samples. In our study, we compared a multiplex ddPCR assay with a singleplex ddPCR assay, and there was no significant difference in quantification or qualification ability. However, any type of PCR system is prone to the misclassification of viral detection due to low sensitivity or specificity of the primer/probe  set. As a result, optimization of the primers and probe becomes critical. Regardless of the PCR type, a multiplex assay can be adopted as a diagnostic tool that quantifies multiple viral targets, while conserving samples in a clinical setting.

## Conclusion

Despite the availability of advanced genomic technology, establishing an association between ubiquitous viruses and primary brain tumors remains challenging. Once again, the present study is a cross-sectional screening of multiple herpesviruses in low- and high-grade astrocytoma specimens. Other detection methods and study designs should be used before making firm conclusions about the association of CMV with glioblastoma.

However, these results have shown that several other herpesviruses may be associated with high-grade astrocytomas. Moreover, HHV-6 and EBV need to be further investigated in other types of primary brain cancers and their possible mechanisms of pathogenesis further explored. Ultimately, blinded and randomized clinical trials of anti-viral agents or other anti-viral intervention strategies may be the only way to describe an association between human herpesviruses and GBM or other cancers.
